# Social support and adaptation to the disease in men and women with psoriasis

**DOI:** 10.1007/s00403-012-1235-3

**Published:** 2012-03-29

**Authors:** Konrad Janowski, Stanisława Steuden, Aldona Pietrzak, Dorota Krasowska, Łukasz Kaczmarek, Ilona Gradus, Grażyna Chodorowska

**Affiliations:** 1 Department of Psychology, University of Finance and Management in Warsaw, ul. Pawia 55, 01-030 Warsaw, Poland; 2 Department of Clinical Psychology, John Paul II Catholic University of Lublin, Al. Racławickie 14, 20-950 Lublin, Poland; 3 Department of Dermatology, Venereology and Pediatric Dermatology, Medical University of Lublin, ul. Radziwiłłowska 13, 20-080 Lublin, Poland

**Keywords:** Social support, Quality of life, Psoriasis, Adaptation to disease, Depressive symptoms, Gender differences

## Abstract

Social support was shown to be an important factor buffering negative effects of stress in a range of clinical populations. Little is known, however, about the role of social support in the population of patients with psoriasis although strong psychosocial stress has been implicated in this disease. The objective of this study was to evaluate the association between social support and selected indices of adaptation to life with the disease, including health-related quality of life, depressive symptoms and acceptance of life with the disease, in a sample of patients with psoriasis. Additionally, gender differences in these relationships were analyzed. One-hundred-four patients with psoriasis completed psychological tests measuring disease-related social support, health-related quality of life, depressive symptoms and acceptance of life with the disease. Psoriasis severity was assessed by Psoriasis Area and Severity Index. The patients reporting higher social support levels had significantly higher quality of life, lower depression levels, and higher acceptance of life with the disease. The strengths of these effects, however, were different in women and men. Higher social support was slightly more closely associated with better acceptance of life with the disease in men than in women. However, higher social support was more closely associated to lower depression and better quality of life in women than in men. Among different types of social support, tangible support was found to be the best predictor for the all adaptation indices. Effects of social support perceived by psoriasis patients on adaptation to the disease may be gender-related and exact pathways of these effects may depend on the type on the dimension of social support and the selected type of adaptation indicator. Tangible support seems the most important type of support contributing to better adaptation in both women and men with psoriasis.

## Introduction

Adaptation to living with a disease is a broad term which encompasses a range of phenomena, including quality of life, emotional well being, good self-esteem, acceptance of life with a disease, social participation and fulfilment of social roles [9, 45]. In chronic diseases, such as psoriasis, affected individuals need to develop psychological mechanisms enabling them to adapt to the disease in the best way possible. Therefore, numerous studies have been undertaken to investigate factors that determine levels of adaptation to living with a chronic disease across a range of medical conditions [20, 50], including psoriasis [13]. Social support is an important aspect of social relationships which is strongly implicated in the process of adaptation to one’s own disease [27]. Social support serves to satisfy the person’s needs, particularly in difficult, critical circumstances, through the relationships with significant persons and reference groups [7, 42, 52].

Several types of social support have been differentiated [4, 26, 34, 48, 53]. In the context of adaptation to disease, Sęk and Cieślak [43] identified five types of social supports: instrumental, informational, tangible, emotional and spiritual. Instrumental support involves provision of advice and other indirect means enabling the person to cope more effectively with a stressful situation. These can be, for example, the instructions on how to cope with a given problem, or how to behave in a situation. Informational support is understood as provision of disease- or health-relevant information for a patient. Such information can be obtained from professional (e.g. doctors, nurses and the scientific literature) or from non-professional sources (e.g. other patients, superstitions) and helps the patient understand better his/her health-related situation. Tangible support consists in obtaining material help, for example financial aid, or other necessary goods. Emotional support involves evoking the feelings and emotional states in the patient which can relieve the patient’s negative affect and through this facilitate coping with the disease on the emotional level. Spiritual support involves those actions of other people (e.g. a minister, a prayer group) which help the patient to attribute a deeper, spiritual meaning to their disease or suffering. Globally, thus, disease-related social support is the amount of emotional and spiritual care, tangible and instrumental aid, and information, perceived as available for an individual when coping with the disease [43].

In contrast to other clinical populations, strikingly few studies investigated the role of social support in patients with psoriasis [32, 37]. This seems odd, since social factors have been reported as particularly essential in adaptation to this disease. For instance, social stigma and social rejection were shown to exert strong negative impact on psychosocial functioning in psoriasis patients [18, 19, 22]. Additionally, living with psoriasis was linked to high psychosocial stress, and social domains of quality of life were reported as remarkably strongly compromised in these patients [29]. Disruptions of social interactions, even with the close ones, were also reported to occur due to psoriasis [1]. Therefore, it seems plausible that provision of social support may be of particular importance for this group of patients, which has already been suggested by a recent study on support from online psoriasis communities [28].

Still less is known about potential gender differences in the links between social support and adaptation to the disease. Gender was a rarely controlled variable in such studies even though general social support research indicated the women had wider social networks than men, had more psychosocial resources available to them [15], and reported higher levels of perceived social support then men [51]. To our knowledge, no studies so far investigated gender differences in the impact of social support on adaptation to psoriasis.

The objective of our study was to evaluate the associations between perceived social support and selected indices of adaptation to the disease in patients with psoriasis. We hypothesised that social support could have impact on such indices of adaptation as health-related quality of life, acceptance of life with the disease and depressive symptoms. We also undertook to investigate gender differences in the associations between social support and the indices of adaptation to life with psoriasis. Additionally, we controlled for the effects between psoriasis-related clinical variables on the one hand, and social support and other psychosocial variables on the other hand.

## Materials and methods

### Participants

Overall, 113 patients with psoriasis took part in the study. The recruitment procedure involved approaching alternately each consecutive male and female patient admitted to the Dermatology Clinic, to ensure equal sex distribution across the sample. Data from nine patients were excluded due to a failure in completing the questionnaires or sizeable missing data. All patients were thoroughly informed about the goal and conditions of the participation in the study and written informed consent for participation was obtained from all patients. The final study sample consisted of 104 patients with psoriasis vulgaris who were hospitalised between November 2007 and May 2008 at the Dermatology Clinic in Lublin, Poland. Fifty-two were males and 52 were females.

Upon enrollment, information was collected for each patient on age, gender, marital status, education, employment status, disease duration, occurrence of lesions on readily visible versus possible to cover body areas, number of hospitalizations, co-existence of other diseases and residence with or without family. Severity of the illness was estimated by means of the Psoriasis Area and Severity Index (PASI) and percent of lesional skin. PASI is a well-established standard measure of psoriasis severity combining the severity of psoriasis symptoms (erythema, infiltration and desquamation) and percent of lesional skin into one numerical index [14]. The evaluation of the disease severity was always done by one researcher (G. Ch.) to avoid inter-rater error variance.

### Psychological testing

Each patient was requested to fill in a battery of psychological questionnaires. The questionnaires were distributed by one of three researchers (A.P., I.G. and Ł.K.) to the patients during their stay at the ward. The researcher provided the patient with a standard instruction on how to fill in the questionnaires. The following psychological measures were applied:

Acceptance of Life with the Disease Scale, developed by Janowski and Steuden, is a 20-item self-report questionnaire measuring an overall level of adaptation to the disease, understood as the ability to reconcile with the disease and retain overall satisfaction with life in spite of the disease burden. People who score high on this scale are also able to distance themselves from their disease and its symptoms. In the standardisation study, reliability of this instrument was found to be high: Cronbach’s alpha = 0.91 and split-half reliability = 0.94. Factor analysis yielded three factors: (1) satisfaction with life (nine items, Cronbach’s alpha = 0.90), (2) reconcilement with the disease (six items, Cronbach’s alpha = 0.80) and (3) self-distancing from the disease (five items; Cronbach’s alpha = 0.69). Respondents answer to the statements on a 4-grade scale, ranging from 1 to 4. The total scores can theoretically range from 20 to 80, with higher scores indicating greater acceptance of life with the disease. The full contents of this instrument are in the Appendix.

Disease-Related Social Support Scale, developed by Brachowicz, Janowski and Sadowska [6], consists of 30 self-report items developed to measure perceived levels of social support available for the patient over the period of their being ill. The scale consists of five subscales corresponding to five types of social support distinguished by Sęk and Cieślak [43]: instrumental, informational, tangible, emotional, and spiritual support. All subscales composed of six items and have satisfactory reliabilities: (1) spiritual support (Cronbach’s alpha = 0.87), (2) instrumental support (Cronbach’s alpha = 0.85), (3) informational support (Cronbach’s alpha = 0.86), (4) tangible support (Cronbach’s alpha = 0.84) and (5) emotional support (Cronbach’s alpha = 0.86). The total score, indicative of the overall perceived support, is a sum of the scores from the subscales, with Cronbach’s alpha = 0.94 and split-half reliability = 0.92. Respondents provided answers to the statements on a 4-grade scale, with higher scores suggesting higher levels of perceived social support. Exemplary items are given below:

*I am informed about my health state, as I need it.*

*There are people who teach me how to solve problems.*

*There are people willing to give me financial support when I need it.*

*Other people console me if I need it.*

*There are people who assist me with searching for meaning of life in my disease.*

Skindex-29 is a self-report questionnaire developed by Chren et al. [8] measuring health-related quality of life in patients with skin diseases. The instrument consists of three subscales: physical symptoms, functioning and emotions. The patients respond on a 5-point scale, higher scores indicate poorer quality of life. The theoretical range of the scores is from 29 to 145. The questionnaire had been adapted and validated in the Polish sample with resulting very good psychometric properties resembling those reported for the original American version, with Cronbach’s reliability coefficient for the total score alpha = 0.96. The reliability coefficients for the subscales are as follows: physical symptoms (Cronbach’s alpha = 0.80), functioning (Cronbach’s alpha = 0.92) and emotions (Cronbach’s alpha = 0.93) [46]. Physical symptoms cover different subjective complaints related to skin, such as burning, itching, bleeding, or pain. The functioning scale measures the negative impact of a skin disease on different everyday activities, including quality of sleep, work, hobby, social relationships, closeness to others and self-exclusion from social participation. Emotions include subjective emotional experience of fears, depression, worries about appearance, anger, embarrassment, frustration, humiliation and irritation.

Beck Depression Inventory (BDI): is one of the most commonly world-wide used self-report instruments measuring levels of depressive symptomatology. The inventory consists of 21 categories of depressive symptoms, and the subject is requested to select the response within each category that describes best his/her mental condition. BDI yields the scores ranging theoretically from 0 to 63, with higher scores indicating more severe depressive symptomatology. The usual threshold for detection of depression is the score of 12 [2, 3]. Most of the researchers report alpha coefficients higher than 0.75, the average coefficient for psychiatric samples amounts to 0.88, and for the nonpsychiatric samples is 0.82 [41].

### Statistical analyses

Descriptive statistics are presented as mean and standard deviations (*M* ± SD). Smirnoff–Kolgomorov test was used to check for normal distribution of the variables. Since the distribution of the disease-related social support scale scores was significantly different from the normal distribution, non-parametric statistical tests were applied in further analyses. Spearman’s rho correlations were calculated to assess associations between social support and other psychological variables. The correlations were calculated separately for men, women and for the total sample. Regression analysis was applied to explain the participation of particular social support dimensions in accounting for the variance in the indices of adaptation to the disease (acceptance of life with the disease, depressive symptoms, and quality of life). Cluster analysis (*k* means method) was used to identify the subgroups of patients homogeneous with respect to overall adaptation to disease, and analysis of variance was applied to compare the mean scores on social support dimensions between the identified subgroups. The level of statistical significance was set at 0.05.

## Results

### Clinical and psychological characteristics of the sample

At the time of the study, subjects were aged between 15 and 73 (*M* = 45.6, SD = 14.24). The disease duration ranged from 1 month to 52 years (*M* = 18.68, SD = 13.31). The scores on the PASI scale were in the range of 7 to 43.5 and were indicative of moderate to severe psoriasis. The mean PASI score was 24.04 (SD = 5.34). The percent of lesional skin ranged from 0.5 to 75 % (*M* = 30.81, SD = 14.12). The life-time number of previous hospitalizations due to psoriasis was from 0 to 65 (*M* = 10.38, SD = 13.79). Skin lesions were located on uncovered body areas (head, face, neck and hands) in 75 % of the patients, and on body parts possible to cover with clothes in 25 % of the patients. Eighty-nine percent of patients reported living with family, and 11 % lived alone.

No statistically significant gender differences were found for social support or acceptance of life with the disease. Statistically significant differences were observed between men and women only on depressive symptoms (Mann–Whitney test *z* = −2.27, *P* = 0.018) and on the emotions dimension of quality of life (Mann–Whitney test *z* = −2.02, *P* = 0.044), where women scored significantly higher than men, suggesting more severe depressive symptoms and worse quality of life in the domain of emotions.

### Psoriasis severity and psychosocial variables

The clinical characteristics of psoriasis severity (the PASI score, number of hospitalizations, percent of lesional skin and disease duration) were found to be unrelated to depressive symptoms (BDI score) both in the total sample and in the samples of men and women. Similarly, no statistically significant correlations were observed between severity of psoriasis and acceptance of life with the disease in either sample. In the total sample, out of psoriasis severity indices, only disease duration was significantly negatively correlated with one dimension of social support—instrumental support (*r* = −0.20), *P* = 0.044). In the total sample, the percent of lesional skin was correlated with the global index of quality of life (*r* = 0.27, *P* = 0.006), functioning (*r* = 0.30, *P* = 0.002), and emotions (*r* = 0.23, *P* = 0.019), and PASI was correlated with functioning (*r* = 0.27, *P* = 0.033). In females, disease duration correlated with instrumental support (*r* = −0.30, *P* = 0.030) and with physical symptoms (*r* = 0.28, *P* = 0.045). In males, PASI was significantly related to quality of life: global index (*r* = 0.32, *P* = 0.030), functioning (*r* = 0.35, *P* = 0.016) and emotions (*r* = 0.34, *P* = 0.020). In males, also percent of lesional skin was correlated with the same indices of quality of life: global index (*r* = 0.40, *P* = 0.003), functioning (*r* = 0.40, *P* = 0.003) and emotions (*r* = 0.39, *P* = 0.005).

### Social support and adaptation to the disease—correlational analysis

The global level of perceived social support was found to be significantly positively related to acceptance of the disease in the whole sample, and both in men and women. The association between these variables was slightly stronger for men than for women. All dimensions of social support were statistically significantly related to acceptance of the disease, when calculated for the whole sample. Considerable differences between men and women were uncovered with respect to the correlations of tangible and emotional support with acceptance of the disease: these associations were much stronger for men (Table 1).

**Table 1:** Spearman’s rho correlations between social support, and acceptance of the disease and depressive symptoms

Social support dimensions	Sample	Acceptance of life with the disease	Depressive symptoms	Health-related quality of life			
				Global score	Physical symptoms	Functioning	Emotions
Global support	Total	0.40***	−0.36***	−0.32***	−0.28**	−0.33***	−0.26**
	Men	0.46***	−0.24	−0.26	−0.34*	−0.24	−0.19
	Women	0.35**	−0.48***	−0.37**	−0.23	−0.40**	−0.32*
Spiritual support	Total	0.33***	−0.30**	−0.25*	−0.21*	−0.28**	−0.19
	Men	0.37**	−0.27	−0.28*	−0.31*	−0.31*	−0.16
	Women	0.27*	−0.30*	−0.19	−0.07	−0.22	−0.17
Instrumental support	Total	0.32***	−0.28**	−0.26**	−0.22*	−0.27**	−0.18
	Men	0.32*	−0.17	−0.20	−0.31*	−0.16	−0.11
	Women	0.34*	−0.39**	−0.33*	−0.14	−0.38**	−0.26
Informational support	Total	0.27**	−0.24*	−0.17	−0.15	−0.15	−0.17
	Men	0.26	−0.08	−0.01	−0.13	0.04	−0.03
	Women	0.28*	−0.42**	−0.35**	−0.25	−0.35*	−0.30*
Tangible support	Total	0.44***	−0.45***	−0.42***	−0.36***	−0.39***	−0.38***
	Men	0.57***	−0.38**	−0.33*	−0.32*	−0.29*	−0.32*
	Women	0.30*	−0.50***	−0.47***	−0.37**	−0.45***	−0.44***
Emotional support	Total	0.32***	−0.28**	−0.25**	−0.22*	−0.28**	−0.18
	Men	0.40**	−0.28*	−0.28*	−0.30*	−0.26	−0.20
	Women	0.25	−0.26	−0.24	−0.14	−0.29*	−0.16

* *P* ≤ 0.05

** *P* ≤ 0.01

*** *P* ≤ 0.001

The global index of social support was found to be significantly negatively related to levels of depressive symptomatology when analyzed in the whole sample and in the women group. This association, however, was found statistically insignificant for men. The correlations between particular dimensions of social support and depressive symptoms were generally stronger for women than for men (except for emotional support). The strongest association was observed between tangible support and depressive symptoms: for the total sample and both men and women. The most noticeable differences between men and women were found with respect to the impact of informational and instrumental support on depression, with these links significantly stronger for women than for men (Table 1).

The global index of social support was found to be significantly inversely correlated with the global index of quality of life in the whole sample, indicating that higher levels of perceived social support tend to co-occur with lower decrements in health-related quality of life. This tendency was also true for women but not for men. In men, the relationship between the global index of social support and quality of life was statistically insignificant. The strongest associations were observed between the dimension of tangible support and quality of life, and this was true both for women and men, although the strength of the relationship was considerably greater in women. Some interesting gender differences were also found with respect to spiritual, informational and emotional support (Table 1).

### Social support and adaptation to the disease—regression analysis

Regression analysis was applied to identify social support dimensions most significantly accounting for the variance in acceptance of life with the disease, depressive symptoms and quality of life. We carried out a series of regression analyses separately for each index of adaptation to the disease. In each case, we first entered gender as an independent variable (males were coded as 0 and females as 1) and in the second block all dimensions of social support were entered. In this way, we could control separately for the effects of gender and each factor of social support. The obtained results of the regression analyses are presented in Table 2.

**Table 2:** The stepwise regression analysis models explaining the variance in the indices of adaptation to life with psoriasis

Variables	*B*	Standard error of *B*	Beta	*t*	*P*
*Acceptance of life with the disease (total score)*					
*R* = 0.438, *R* ^2^ = 0.192, corrected *R* ^2^ = 0.176, *F* = 21.635, *P* = 0.000					
Constant	46.87	4.74		9.88	0.000
Gender	−2.63	1.95	−0.12	−1.35	0.180
Tangible support	0.96	0.21	0.42	4.65	0.000
*Depressive symptoms (BDI total score)*					
*R* = 0.479, *R* ^2^ = 0.230, corrected *R* ^2^ = 0.214, *F* = 22.154, *P* = 0.000					
Constant	21.24	4.00		5.31	0.000
Gender	4.35	1.64	0.23	2.65	0.009
Tangible support	−0.82	0.17	−0.41	−4.71	0.000
*Quality of life (Skindex-29)*					
*Total score*					
*R* = 0.438, *R* ^2^ = 0.192, corrected *R* ^2^ = 0.176, *F* = 22.363, *P* = 0.000					
Constant	117.97	10.00		11.80	0.000
Gender	4.56	4.11	0.10	1.11	0.270
Tangible support	−2.06	0.44	−0.42	−4.73	0.000
*Emotions*					
*R* = 0.443, *R* ^2^ = 0.197, corrected *R* ^2^ = 0.181, *F* = 19.774, *P* = 0.000					
Constant	39.94	3.97		10.07	0.000
Gender	3.37	1.63	0.19	2.07	0.041
Tangible support	−0.77	0.17	−0.40	−4.44	0.000

The global index of acceptance of life with psoriasis was statistically significantly accounted for only by tangible support (approximately 19 % of explained variance). Gender turned out to be insignificant both alone and in combination with social support dimensions. For two dimensions of acceptance of life with the disease: reconcilement with the disease and self-distancing from the disease—the same finding was obtained, namely, in both cases only tangible support but not gender was found to be statistically significant. In the case of satisfaction with life, the percent of explained variance was higher (approximately 28 %) and two dimensions of social support reached statistical significance, namely tangible support and informational support. Tangible support was found to be associated with the global score of acceptance of life with the disease positively, and similarly, informational support was positively linked to satisfaction with life, which means that higher levels of perceived tangible and informational support are predictor of better acceptance of life with the disease (Table 2).

Regression analysis for depressive symptoms revealed that both gender and tangible support were statistically significant in accounting for the variance of this variable and they explained together 23 % of variance. The analysis revealed that the female gender was predictive of higher depressive symptoms whereas higher tangible support levels were related to lower depressive symptoms. Other social support dimensions did not reach the statistical significance level.

Regression analysis for quality of life indicated that tangible support was the only support factor significantly accounting for the variance in all indices of quality of life (the global score, physical symptoms, functioning and emotions). For the global quality of life score, the regression model accounted for approximately 19 % of the variance. In the quality of life domain of emotions, gender was found as a second independent variable significantly contributing to the variance in this variable (approximately 20 % of explained variance). For the domains of physical symptoms and functioning, tangible support was the only variable contributing significantly to the variance. Higher scores on the quality of life scale (lower quality of life) were associated with lower tangible support, and in the case of emotions, female gender was related to lower quality of life (Table 2).

### Social support and adaptation to the disease—cluster analysis

**Fig. 1:**
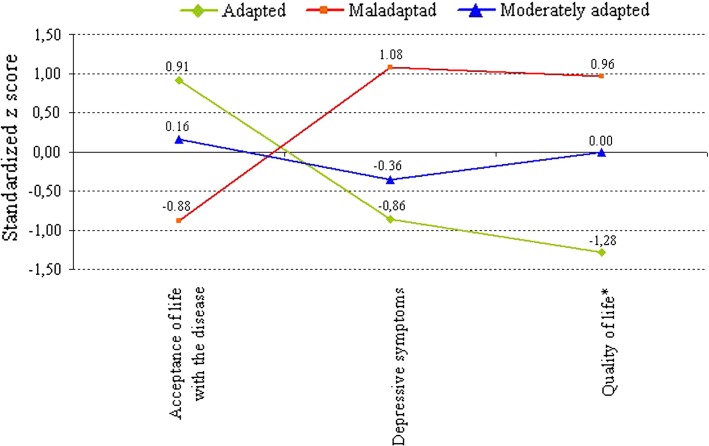
Mean profiles of adaptation indices for three groups of psoriasis patients identified in cluster analysis (Adapted, *N* = 26; moderately adapted, *N* = 43; maladpated, *N* = 35). The total scores on Acceptance of Life with the Disease Scale, Beck depression inventory and Skindex were the basis for the discrimination of the groups. * Higher scores are indicative of lower quality of life

In the next step of the analysis, we proceeded to distinguish the subgroups of psoriasis patients differing with respect to their overall adaptation, taking into account simultaneously all three indices of adaptation. We applied hierarchical cluster analysis (the *k* means method) on the global scores from Acceptance of Life with the Disease Scale (acceptance), Beck Depression Inventory (depressive symptoms) and Skindex (quality of life). As a result, we obtained three different subgroups of patients which we labelled adapted (*N* = 26), moderately adapted (*N* = 43) and maladapted *N* = 35). The mean profiles for the subgroups are presented in Fig. 1. Before entering the data into cluster analysis, we linearly transformed the scores on the three scales into standardised *z* scores, to enable their direct comparison.

The adapted group was characterised by the highest scores on acceptance of life with the disease, lowest on depressive symptoms and had highest levels of quality of life (low scores on Skindex indicate high quality of life). The moderately adapted group showed average levels of both acceptance of life with the disease, depressive symptoms and quality of life. The maladapted group scored lowest on acceptance of life with the disease, highest on depressive symptoms and revealed the lowest levels of quality of life (high scores on Skindex).

The identified subgroups were then compared on their reported levels of social support dimensions (Table 3). Analysis of variance evidenced statistically significant differences between the compared groups for all dimensions of social support, with an exception for emotional support where only a statistical trend was observed (*P* = 0.097). The subgroups with higher levels of overall adaptation to the disease also reported higher levels of social support. Post hoc tests showed that the most pronounced differences were observed for the adapted and maladapted subgroups, no statistical differences were found, however, between the adapted and moderately adapted subgroups. The most significant differences were observed for tangible support (*F* = 9.13) and global support (*F* = 6.01).

**Table 3:** Mean scores, standard deviations and differences between the groups of adapted, moderately adapted and maladapted patients on perceived social support

Social support	Groups							
	Adapted (*N* = 26)		Moderately adapted (*N* = 43)		Maladapted (*N* = 35)		ANOVA	
	*M*	SD	*M*	SD	*M*	SD	*F*	*P*
Global support	96.85	17.26	92.19	16.65	82.37	17.15	6.01^a.b^	0.003
Spiritual support	19.96	3.77	18.49	4.28	17.09	4.69	3.35^a^	0.039
Instrumental support	18.19	4.04	17.30	3.94	15.31	4.34	4.11^a.b^	0.019
Informational support	19.85	3.55	19.91	3.55	17.91	3.42	3.66^a.b^	0.029
Tangible support	19.19	4.09	17.56	4.24	14.51	4.81	9.13^a.b^	0.000
Emotional support	19.65	4.3	18.93	3.61	17.54	3.91	2.39^a^	0.097

Statistically significant differences between the following groups

^a^Adapted–maladapted

^b^Moderately adapted–maladapted

## Discussion

The objective of this study was to explore the associations between social support and selected indices of adaptation of the disease in patients with psoriasis. Gender differences in these relationships were also addressed. The results of the initial analyses showed that the women and men with psoriasis reported similar levels of perceived social support. This finding is somewhat different from some other studies which indicated that women tend to perceive higher levels of social support than men [15, 51].

The findings from the correlational analysis showed that higher levels of global perceived social support were related to better acceptance of life with the disease in both men and women with psoriasis. Among different dimensions of social support, tangible support was found to be most closely related to better acceptance of life with the disease in the whole group, and this was particularly significant for men. This strong link can be probably accounted for by essential importance attributed to material security by patients with psoriasis. Some studies emphasised a burdensome impact of psoriasis on economic quality of life [12, 31] which may explain why tangible support proved to be most closely related to acceptance of life with psoriasis. Interesting gender differences were found with respect to three dimensions of social support: spiritual, tangible and emotional support. These social support dimensions were clearly more closely related to better acceptance of life with psoriasis in men than in women. These differences may result from the fact that men’s satisfaction with life, including the life with psoriasis, is dependent on social acceptance, social recognition and material status to a greater extent than that of women’s [39]. It should be mentioned that one study found that gender had no effect on acceptance of psoriasis [54] although this study did not explore the impact of gender on the relationship between social support and acceptance of the disease.

Higher global levels of social support were also significantly correlated with lower depressive symptoms, although this correlation was found to be statistically significant only in women. Therefore, social support may be viewed as a buffer for emotional disturbances revealed under the form of depressive symptomatology in women with psoriasis, whereas this effect may be insignificant for men with psoriasis. The differences within this effect between men and women were particularly strong for instrumental and informational support, which suggests that women profit better from these types of support in terms of prevention of depressive symptoms. In one study, social support was reported to be related to lower depression even irrespective of gender [16]. However, our findings linking higher social support with lower depression in women whereas to a lesser degree in men are corroborated by findings from some other studies. For instance, gender differences were reported with respect to anxiety in a study on the influence of social support on adaptation to surgery. Women declaring high informational support exhibited lower anxiety levels, whereas men showed low anxiety even when deprived of informational support [30]. We hypothesise that informational and instrumental types of support may make women, but not men, feel more in control of the situation, increase the controllability of the disease as a stressor, reduce helplessness, which in turn diminishes the risk of development of depressive symptoms. Similar explanations were proposed for patients with major depressive disorder and dysthymia [10].

It is of note that tangible and emotional supports were the only social support dimensions that were significantly related to lower depressive symptoms in men, and the same two dimensions were important for men with respect to better acceptance of the disease. This suggests that these two social support dimensions correspond with two levels of sense of security in men: tangible and emotional security. We believe that the presence of tangible and emotional support favours better acceptance of life with the disease and buffers development of depressive symptoms in men. In contrast, all types of social support buffer depressive symptoms in women. Therefore, disturbed access to one social support dimension may be less detrimental for women because it may be more easily compensated by profiting from other remaining accessible social support dimensions. In line with our explanatory hypotheses, some other researchers also reported that social support had a stronger buffering effect on depressive symptoms in females than in males [33]. Similarly, Hann et al. [23] also reported that in a sample of cancer patients, larger social support network was associated with less severe depression for women but not for men.

Gender differences in the relationship between social support and depressive symptoms implicate that social support will be generally more effective in prevention of depression for women than for men with psoriasis; therefore, psychosocial interventions for psoriasis patients should be gender specific. It is of particular importance in light of data from other studies that women from various clinical populations, including psoriasis patients, generally score higher on depression measures than men [5]. Therefore, provision of instrumental and informational support for patients with psoriasis, especially for women, should be taken into consideration when planning psychosocial education. In contrast, men should be sensitised on how to utilise instrumental and informational support in order to optimise their adaptation to psoriasis.

Some studies reported that men had higher health-related quality of life than women [17] although in our study patients with psoriasis did not show gender differences on the global index of quality of life. However, men did show better quality of life than women in the domain of emotions. The analysis of correlations between social support and quality of life showed that higher global index of support was related to higher quality of life in the total sample and in women, but not in men. When analyzing individual dimensions of support it was found that the global index of quality of life was related to instrumental and informational support in women but not in men. Global quality of life was correlated with emotional and spiritual support in men but not women, whereas with tangible support both in women and men. Again, among all support dimensions, the strongest relationship to quality of life was observed for tangible support. It is also of note that informational support was relatively strongly related to quality of life in women but it was entirely insignificant for quality of life in men. Therefore, it can generally be claimed that quality of life in patients with psoriasis is related to perceived social support but this relationship is also mediated by gender, and the protective effects of support on quality of life are generally more noticeable in women. Our findings differ somewhat from those reported by Gallicchio et al. [17] who observed that health-related quality of life in a community-based sample was correlated with social support but this relationship was not mediated by gender. The differences between the latter and our study can, however, be attributed to a different type of sample (clinical vs. community based) and to a different conceptualization of social support (functional vs. structural approach).

The findings from regression analyses we conducted for the indices of acceptance of life with the disease, depressive symptoms and quality of life confirmed again the most critical role of tangible support for all these indices of adaptation to psoriasis. Some other studies also indicated the importance of tangible support for adaptation to a chronic disease. Symister and Friend [47] reported that tangible support was an important predictor of an increase in optimism in a sample of patients with renal failure. In another study, tangible support together with self-efficacy was independent predictors of mental health status in patients with myasthenia gravis [40].

On the basis of cluster analysis, three types of patients were identified with different levels of overall adaptation to psoriasis: adapted, moderately adapted and maladapted. It is of note that this classification combined the global scores on all the applied measures of adaptation to the disease. Therefore, this classification probably corresponds best to overall adaptation of these patients to living with psoriasis. When compared on the dimensions of support, these subgroups were found to differ significantly on all dimensions of social support. Two observations seem particularly interesting. First, the groups with decreasing levels of adaptation presented systematically lowered levels of perceived social support. Second, tangible support and global support were the most strongly differentiating factors although the most pronounced differences between the subgroups were again found for tangible support. The general conclusion from this part of analysis is that when the indices of adaptation to the disease are combined, all dimensions of support reach the significance level.

## Conclusion

Certain previous studies reported that women were more inclined than men to look for social support [21, 25, 35, 44] and to benefit from social support when coping with stress [11, 38]. In the light of our findings, the overall picture seems a little more complex. We have demonstrated that social support may exert diverse impact on different indices of adaptation to the disease and these effects are additionally mediated by gender.

In the case of our patients with psoriasis, gender differences in adaptation to the disease may be accounted for also by social factors influencing self-perception and social appearance in particular. Social, psychological, cultural and biological factors contribute to the concept of gender, and these factors may influence illness behaviour [24, 36, 49]. Psoriasis is an illness closely related to appearance concerns, and therefore, the buffering impact of social support on depression and quality of life in women with psoriasis may be mediated through a decrease in appearance concerns and increase in feelings of social acceptance. In future, further studies should probably pay more attention to the social context of this disease to unravel the exact nature of the relationship between social support and adaptation to psoriasis.

Generally, the findings from this study confirm previous results indicating that higher social support may have beneficial effects on adaptation to life with the disease. What is more interesting, however, our study emphasises that the strength of these effects may be gender related. Additionally, gender differences regarding the impact of social support on adaptation to life with a chronic disease, such as psoriasis, may strongly depend on the type of selected indicator of adaptation. A detailed analysis of the mutual relationships between different dimensions of social support and different indicators of adaptation to life with the disease can reveal specific pathways through which social support affects how well men and women adapt to their chronic disease.

## References

[1] Basra MK, Finlay AY (2007). The family impact of skin diseases: the Greater Patient concept. Br J Dermatol.

[2] Beck AT, Ward CH, Mendelson M, Mock J, Erbaugh J (1961). An inventory for measuring depression. Arch Gen Psychiatry.

[3] Beck AT, Steer RA, Brown GK (1996). Beck depression inventory-second edition manual.

[4] Borgatti SP, Mehra A, Brass DJ, Labianca G (2009). Network analysis in the social sciences. Science.

[5] Boutin-Foster C, Charlson ME (2007). Do recent life events and social support explain gender differences in depressive symptoms in patients who had percutaneous transluminal coronary angioplasty?. J Womens Health.

[6] Brachowicz M (2008) Psychological factors in coping with infertility-related stress. Unpublished doctoral dissertation, John Paul II Catholic University of Lublin, Lublin, Poland

[7] Caplan G (1981). Mastery of stress: psychosocial aspects. Am J Psychiatry.

[8] Chren MM, Lasek RJ, Flocke SA, Zyzanski SJ (1997). Improved discriminative and evaluative capability of a refined version of Skindex, a quality-of-life instrument for patients with skin diseases. Arch Dermatol.

[9] de Ridder D, Geenen R, Kuijer R, van Middendorp H (2008). Psychological adjustment to chronic disease. Lancet.

[10] Diener C, Kuehner C, Brusniak W, Struve M, Flor H (2009). Effects of stressor controllability on psychophysiological, cognitive and behavioural responses in patients with major depression and dysthymia. Psychol Med.

[11] Etzion D, Pines A (1981) Sex and culture as factors explaining reported coping behavior and burnout of human service professionals. A social psychological perspective. Tel Aviv University, The Israel Institute of Business Research, Tel Aviv

[12] Feldman SR, Fleischer AB, Reboussin DM, Rapp SR, Bradham DD, Exum ML, Clark AR (1997). The economic impact of psoriasis increases with psoriasis severity. J Am Acad Dermatol.

[13] Finzi A, Colombo D, Caputo A, Andreassi L, Chimenti S, Vena G, Simoni L, Sgarbi S, Giannetti A (2007). Psychological distress and coping strategies in patients with psoriasis: the PSYCHAE study. J Eur Acad Dermatol Venereol.

[14] Frederiksson T, Pettersson U (1978). Severe psoriasis—oral therapy with a new retinoid. Dermatologica.

[15] Fry PS (2001). The unique contribution of key existential factors to the prediction of psychological well-being of older adults following spousal loss. Gerontologist.

[16] Gallegos-Carrillo K, Mudgal J, Sanchez-Garcia S, Wagner FA, Gallo JJ, Salmeron J, Garcia-Pena C (2000). Social networks and health-related quality of life: a population based study among older adults. Salud Publica Mex.

[17] Gallicchio L, Hoffman SC, Helzlsour KJ (2007). Relationship between gender, social support, and health-related quality of life in a community-based study in Washington County, Maryland. Qual Life Res.

[18] Ginsburg IH, Link BG (1989). Feelings of stigmatization in patients with psoriasis. J Am Acad Dermatol.

[19] Ginsburg IH, Link BG (1993). Psychosocial consequences of rejection and stigma feelings in psoriasis patients. Int J Dermatol.

[20] Gluckman PD, Hanson MA (2007). Developmental plasticity and human disease: research directions. J Intern Med.

[21] Greenglass E, Schwarzer R, Jakubiec D, Fiksenbaum L, Taubert S (1999) The proactive coping inventory (PCI): a multidimensional research instrument. Paper presented at the 20th Internal conference of the Stress and Anxiety Research Society (STAR), Cracow, Poland

[22] Gupta MA, Gupta AK, Watteel GN (1998). Perceived deprivation of social touch in psoriasis is associated with greater psychologic morbidity: an index of the stigma experience in dermatologic disorders. Cutis.

[23] Hann D, Baker F, Denniston M, Gesme D, Reding D, Flynn T, Kennedy J, Kieltyka RL (2002). The influence of social support on depressive symptoms in cancer patient: age and gender differences. J Psychosom Res.

[24] Hibbard JH, Pope CR (1983). Gender roles, illness orientation and use of medical services. Soc Sci Med.

[25] Hildingh C, Fridlund B (1997). Social network and experiences of social support among women 12 months after their first myocardial infarction. Int J Rehabil Health.

[26] House JS, Kahn RL, Cohen S, Syme SL (1985). Measures and concepts of social support. Social support and health.

[27] House JS, Umberson D, Landis KR (1988). Structures and processes of social support. Annu Rev Sociol.

[28] Idriss SZ, Kvedar JC, Watson AJ (2009). The role of online support communities: benefits of expanded social networks to patients with psoriasis. Arch Dermatol.

[29] Kimball AB, Jacobson C, Weiss S, Vreeland MG, Wu Y (2005). The psychosocial burden of psoriasis. Am J Clin Dermatol.

[30] Krohne HW, Slangen KE (2005). Influence of social support on adaptation to surgery. Health Psychol.

[31] Kulkarni AS, Balkrishnan R, Richmond D, Pearce DJ, Feldman SR (2005). Medication-related factors affecting health care outcomes and costs for patients with psoriasis in the United States. J Am Acad Dermatol.

[32] Laffrey SC, Bailey BJ, Craig KK (1996). Social support and health promotion outcomes of adults with psoriasis. Dermatol Nurs.

[33] Landman-Peeters KM, Hartman CA, van der Pompe G, den Boer JA, Minderaa RB, Ormel J (2005). Gender differences in the relation between social support, problems in parent-offspring communication, and depression and anxiety. Soc Sci Med.

[34] Lazarus RS, Lazarus BN (2006). Coping with aging.

[35] Luckow A, Reifman A, McIntosh DN (1998) Gender differences in coping. A meta-analysis. Poster presented to the annual meeting of the American Psychological Association, San Francisco, CA

[36] Nathanson CA (1975). Illness and the feminine role: a theoretical review. Soc Sci Med.

[37] Picardi A, Mazzotti E, Gaetano P, Cattaruzza MS, Baliva G, Melchi CF, Biondi M, Pasquini P (2005). Stress, social support, emotional regulation, and exacerbation of diffuse plaque psoriasis. Psychosomatics.

[38] Prochaska JO, DiClemente CC, Norcross JC (1992). In search of how people change. Applications to addictive behaviors. Am Psychol.

[39] Quick HE (1998). Gender, employment and retirement quality: a life course approach to the differential experiences of men and women. J Occup Health Psychol.

[40] Raggi A, Leonardi M, Mategazza R, Casale S, Fioravanti G (2010). Social support and self-efficacy in patients with Myasthenia Gravis: a common pathway towards positive health outcomes. Neurol Sci.

[41] Richter P, Wernerb J, Heerleinc A, Krausa A, Sauerd H (1998). On the validity of the beck depression inventory: a review. Psychopathology.

[42] Sarason IG, Sarason BR (2004). Abnormal psychology. The problem of maladaptive behavior.

[43] Sęk H, Cieślak R (2005). Wsparcie społeczne, stres i zdrowie (Social support, stress and health).

[44] Shumaker SA, Hill DR (1991). Gender differences in social support and physical health. Health Psychol.

[45] Stanton AL, Revenson TA, Tennen H (2007). Health psychology: psychological adjustment to chronic disease. Annu Rev Psychol.

[46] Steuden S, Janowski K (2001). Application of the questionnaire Skindex to measure quality of life in patients with psoriasis. Przeg Dermatol.

[47] Symister P, Friend R (2003). The influence of social support and problematic support on optimism and depression in chronic illness: a prospective study evaluating self-esteem as a mediator. Health Psychol.

[48] Vaux A, Harrison D (1985). Support network characteristics associated with support satisfaction and perceived support. Am J Community Psychol.

[49] Verbrugge LM (1985). Gender and health: an update on hypotheses and evidence. J Health Soc Behav.

[50] Weinman JA, Petrie KJ, Petrie KJ, Weinman JA (1997). Perceptions of health and illness. Perceptions of health and illness: current research and applications.

[51] Willhite RK, Niendam TA, Bearden CE, Zinberg J, O’Brien MP, Cannon TD (2008). Gender differences in symptoms, functioning and social support in patients at ultra-high risk for developing a psychotic disorder. Schizophr Res.

[52] Williams P, Barclay L, Schmied V (2004). Defining social support in context: a necessary step in improving research, intervention, and practice. Qual Health Res.

[53] Wills TA, Cohen S, Syme L (1985). Supportive functions of interpersonal relationships. Social support and health.

[54] Zalewska A, Miniszewska J, Chodkiewicz J, Narbutt J (2007). Acceptance of chronic illness in psoriasis vulgaris patients. J Eur Acad Dermatol Venereol.

